# Clinical Trial Designs and Measures in Hereditary Spastic Paraplegias

**DOI:** 10.3389/fneur.2018.01017

**Published:** 2018-12-21

**Authors:** Brian Trummer, Dietrich Haubenberger, Craig Blackstone

**Affiliations:** ^1^Neurogenetics Branch, Clinical Research Program, National Institute of Neurological Disorders and Stroke, National Institutes of Health, Bethesda, MD, United States; ^2^Clinical Trials Unit, Clinical Research Program, National Institute of Neurological Disorders and Stroke, National Institutes of Health, Bethesda, MD, United States

**Keywords:** rating scales, spasticity, therapeutics, neurogenetics, neuromuscular

## Abstract

Hereditary spastic paraplegias (HSPs) are a large group of genetically-diverse neurologic disorders characterized clinically by a common feature of lower extremity spasticity and gait difficulties. Current therapies are predominantly symptomatic, and even then usually provide inadequate relief of symptoms. Going forward, HSP therapeutics development requires a systematic analysis of quantifiable measures and tools to assess treatment response. This review summarizes promising therapeutic targets, assessment measures, and previous clinical trials for the HSPs. Oxidative stress, signaling pathways, microtubule dynamics, and gene rescue/replacement have been proposed as potential treatment targets or modalities. Quantitative evaluation of pre-clinical rodent HSP models emphasize rotarod performance, foot base angle, grip strength, stride length, beam walking, critical speed, and body weight. Clinical measures of HSP in humans include 10-m gait velocity, the Spastic Paraplegia Rating Scale (SPRS), Ashworth Spasticity Scale, Fugl-Meyer Scale, timed up-and-go, and the Gillette Functional Assessment Questionnaire. We conducted a broad search for past clinical trials in HSPs and identified trials that investigated pharmacological agents including atorvastatin, gabapentin, L-threonine, botulinum toxin, dalfampridine, methylphenidate, and baclofen. We provide recommendations for future HSP treatment directions based on these prior research experiences as well as regulatory insight.

## Introduction

Hereditary spastic paraplegias (HSPs) arise from mutations in a large number of different genes, encompassing autosomal dominant, autosomal recessive, X-linked, and mitochondrial inheritances; *de novo* mutations have also been described. Together, these conditions coalesce around a common phenotype comprising a progressive spastic gait disturbance, with or without additional clinical features. HSPs occur in ~1.8 per 100,000 people on average globally, but this prevalence can vary significantly across different populations (1). Pathologically, spasticity in the lower extremities arises from length-dependent degeneration and/or abnormal development of corticospinal axons, which can extend to 1 m in length (2).

Most HSPs have been systematically categorized using the prefix SPG followed by a number—with each number indicating a different genetic locus—in order of identification (SPG1-80, plus others). While these genes encode different proteins, the gene products tend to distribute into a relatively small number of cellular localizations and functions, comprising defects in myelination, mitochondrial function, organelle distribution and morphology, axon pathfinding, axon transport, bone morphogenetic protein signaling, and lipid metabolism. The three most common autosomal dominant forms—SPG4, SPG3A, and SPG31—account for about half of all cases and result from mutations in proteins that bind one another and shape the tubular endoplasmic reticulum (ER). The most common autosomal recessive forms—SPG11 and SPG15—are due to loss-of-function mutations in large proteins that bind one another and function in endolysosomal trafficking and autophagic pathways (3–11).

### Search Strategy

Previously published HSP therapeutic studies were searched for in PubMed using the terms “hereditary spastic paraplegia” and “therapeutic,” which also helped to identify HSP clinical trials. Further identification of HSP clinical trials was conducted through PubMed, utilizing the search terms “hereditary spastic paraplegia” and “hereditary spastic paraparesis” with an article-type filter “clinical trial.” Within PubMed, we also utilized the search term “hereditary spastic paraplegia phase.” In clinicaltrials.gov, we searched for “hereditary spastic paraplegia.” Studies employing interventional therapies that were specifically examined in the HSP population (as assessed by author BT) were also selected for review. Clinical assessment tools are assembled from these prior trials later in this review.

## Results

### Preclinical Development: Therapeutic Targets in HSP

To optimize HSP clinical trial design in the future, it is sensible to consider already published preclinical treatment strategies. Oxidative stress is one potential therapeutic target that has been explored for SPG4, the most common form of HSP. Indeed, zebrafish treated with 2′,7′-dichlorofluorescein diacetate (DCF-DA), used to measure oxidative stress, have increased fluorescence when treated with a morpholino that depletes the SPG4 protein spastin (3). FDA-approved drugs that inhibit oxidative stress and ER stress—such as guanabenz, methylene blue, and *N*-acetyl-cysteine—have been tested in various SPG4 animal models (*C. elegans, Drosophila*, and zebrafish), with some success in improving movement (3). Mitochondrial fission has been also targeted, and inhibitors of the mitochondrial fission GTPase DRP1, specifically mdivi-1, improve neurite outgrowth in induced pluripotent stem cells derived from SPG15 and SPG48 patients (4).

A third target for SPG4 is microtubule dynamics more generally, with both microtubule destabilizing and stabilizing agents being investigated (5). This target seems particularly relevant for SPG4, since spastin functions as a microtubule-severing ATPase. Olfactory neurosphere-derived cells from SPG4 patients show altered levels of acetylated α-tubulin, a marker of stabilized microtubules; tubulin-binding drugs (0.5 nM paclitaxel, 0.5 nM vinblastine, 2 nM epothilone D, and 10 μM noscapine) restore acetylated α-tubulin levels in SPG4 patient-derived cells (6). One group has been developing a robotic method to assay plate wells for the identification of drugs that impact fast transport of mitochondria along microtubules, and they have found that nocodazole accomplishes this task by disrupting microtubules (7). In a *Drosophilia* model for SPG4 cold temperature, which destabilizes microtubules, rescues phenotypes in spastin mutant flies, when chilled during development as well as in adulthood (8).

A fourth mechanism under preclinical study for a variety of HSPs where loss-of-function or haploinsufficiency are postulated is gene rescue. For SPG4, lentiviral expression of M1 or M87 isoforms of spastin (generated via the use of different translational start sites) rescued pathogenic defects in SPG4 neurons, reducing neuronal swelling and increasing length of axons (9). In a knock out mouse model of SPG7, adeno-associated virus-mediated intramuscular delivery of the *Spg7* gene product paraplegin improved performance as assessed by rotarod (10). An alternative approach to SPG4 spastin rescue involves knocking down miRNAs that negatively regulate spastin expression, such as MiR-96 and miR-182 (11). Increasing levels of transcription factors such as SOX11 and NRF1 that positively regulate spastin has also been proposed, particularly for NRF1, as elephants with long corticospinal tracts have lost the SOX11 site, which has been replaced by four putative NRF1 binding sites (11).

Additionally, for some autosomal recessive forms of HSP such as SPG11, SPG15, and SPG48, which are characterized by dysfunction in autophagy and endolysosomal pathways, compounds that induce autophagy have been suggested as treatment, such as carbamazepine, rapamycin, among others (12). SPG5, which results from an accumulation of 27-hydroxycholesterol (27-OHC), has been evaluated for responses to cholesterol-lowering drugs (13–15).

### Preclinical Development: Animal Assessment Measures for Therapeutic Efficacy

A variety of animal models for different HSPs, as well as protocols to test physical function in these animals, have been developed. Morpholinos against 14 HSP human genes in zebrafish impact motor neurons and impair locomotion (16). Rodent models have also been developed, along with ways to assess physical function.

One popular measure for rodent models is rotarod performance. *Spg7*^−/−^ mice were placed on a rotating rod with starting speed of 4 rpm, achieving a final speed of 40 rpm in 5 min, with 3–4 trials a day; animals were permitted on the rod for a maximum of 600 s (17). *Spg20*^−/−^ mice at 4–7 months of age were assessed on a rotarod treadmill 3.2 cm in diameter and monitored in three independent trials separated by 15 min for recovery, increasing from 4 to 40 rpm for a maximum of 300 s (18).

A second measure developed to assess mouse HSP models is the foot base angle. *Reep1*^−/−^ knockout mice had the change in foot-base angle over time measured (19). In *Spg15* knockout mice at 8 months of age, there was no difference in body weight or gait abnormality in the knockout mice, but at 12 months knockout mice had a progressive gait disorder quantified by discernable foot base angle changes; by 16 months foot-base angle was reduced to 50° as compared to 75° in wild-type mice (20).

A third measure to assess HSP mouse models is grip strength. In *Spg20*^−/−^ mice, grip strength was measured using a Bioseb meter assessing the maximum force exerted by hind limbs (18).

A fourth measure is beam walking. In a *Spg21* knockout mouse model, male mice from 2 to 12 months were placed on a beam 80 cm long and 1 cm wide; time to walk across the elevated beam was recorded along with the number of foot slips (counted with a tally counter) (21). An alternative measure for beam walking is the number of times a mouse falls off a beam, as used in a *Spg15* knockout mouse model (20).

A fifth measure is the determination of critical speed. Critical speed is measured by running mice on a treadmill with a shock grid delivering 0.2 mA, placed 10 cm from the rear of the cell, to provide a stimulus to make the animals run. There are four runs leading to exhaustion, and a single trial performed each day over 4 days with mice running 18–41 m/min, limited to 45 min or exhaustion as defined by a total number of 50 shocks. Distance (m) is plotted on the y-axis and time(s) on x-axis, and the slope is the critical speed (22). In 4, 12, and 22-month old SPG4 mice, a statistically significant 35% decrease in critical speed was described in mutant mice from 15 to 24 months age, compared to a 17% decrease in control mice (23).

### Clinical Development: Potential Human Assessment Measures for Therapeutic Efficacy

#### Clinical Rating Scales

A clinical rating scale that has been developed specifically for HSPs is the clinical Spastic Paraplegia Rating Scale (SPRS), which comprises 13 items to assess the level of spasticity and shows high internal consistency (24). Changes in SPRS (24, 25) were used as a secondary outcome in a clinical trial investigating the efficacy of botulinum toxin type-A for HSP (26). A subset of this SPRS scale has been used to generate a “spastic subscore,” which is the sum of point values for spasticity of hip adductor muscles, weakness of hip abduction, spasticity of knee flexion and weakness of foot dorsiflexion (27). Some trials have used a 10-m maximum or comfortable gait velocity as a primary outcome measure. Clinicians measured the change in 10-m maximum gait velocity (28, 29) 8 weeks after botulinum toxin type-A injections, taking the average of three trials (26). The Ashworth spasticity scale (30) has been commonly used in clinical trials of HSP. One group has used the walking component of the Gillette Functional Assessment Questionnaire (31) in a baclofen pump trial (32). Also mentioned in single trials are: the Fugl-Meyer scale, which assesses motor function, some sensation, balance and joint function (33); timed up-and-go testing, which tests time to get up from an arm chair, walk 3 m, turn, walk back and sit down (34); multiple sclerosis (MS) impairment scale, which includes a neurological exam and limited neuropsychological tests (35); the Medical Research Council (MRC) scale; brief pain inventory; and fatigue scales. A simple 4 stage rating scale has also been proposed: (i) mild symptoms walking without an aid; (ii) walking without aid but unable to run; (iii) walking with aid; and (iv) wheelchair dependent (36). This 4-stage rating scale was found to predict SPRS scores with *p* < 0.0001 and was inversely related with SF-36 scale at −0.248 with *p* = 0.032 (37).

#### Human Laboratory and Biopsy Biomarkers

Genetic testing has dramatically advanced, and testing patients with physical findings suspicious for HSP is increasingly available at reasonable cost. Currently, >50 genes can be readily tested using comprehensive, commercial HSP panels. A number of exploratory laboratory measures can be pursued to further knowledge of specific variants of HSP. For instance, SPG5 patients with mutations in the CYP7B1 enzyme have a buildup of toxic cholesterol products. Measuring 25-OHC and 27-OHC, as well as their ratio to cholesterol, is 100% sensitive and specific for SPG5 as compared to normal controls (14).

Preliminary work has emphasized miRNAs, specifically miR-140 and miR-691 for SPG31 (38) as well as miR-638 (host gene *DNM2*) (39). One group has described an antibody against amino acid residues 244-326 of *MUDENG* (mu-2-related death-inducing gene, MuD) that may play a role in endosomal protein trafficking, as a biomarker for HSP (40). Another group studying SPG10 obtained skin biopsy samples from the distal leg, reporting a patient with decreased and fragmented staining with anti-synaptophysin, and on electronic microscopy, decreased number of pre-synaptic vesicles (41).

#### Clinical Imaging Tools

Imaging modalities are specific to certain SPGs and not generalizable to all HSPs. One group found that 3 patients with SPG5 had diffuse white matter hyperintensities in the centrum semiovale and corona radiata, while sparing the corpus callosum (42). Additionally, many SPG11 and SPG15 patients have a thin corpus callosum and “ears of the lynx” white matter changes on brain MRI (43). In a study of 3 patients with SPG5, PET scans using ^18^F-fluoro-deoxyglucose showed cerebellar hypometabolism in one of the patients, predominantly affecting the vermis (42). SPG11 patients with paraparesis had decreased paracentral and thalamic metabolism (43). Utilization of proton magnetic resonance spectroscopy has found that in SPG4, the choline/creatine (Cho/Cr) ratio in the motor cortex was significantly lower than in controls (*p* = 0.047), possibly indicating reduced membrane turnover or else cell loss (44).

In SPG54 due to mutation in *DDHD2*, a pathologic spectral lipid peak at 1.3 ppm was identified. The DDHD2 gene product normally hydrolyzes phosphatidic acid and has activity toward other phospholipids including phosphatidylethanolamine, acting typically at the ER-Golgi intermediate compartment (45). Finally, in patients with complicated SPG7, optical coherence tomography (OCT) has been useful in finding retinal nerve fiber layer thinning, likely due to mitochondrial defects (46). All SPG7 patients had pathologic OCT, but only 40% had decreased visual acuity and 50% had optic disc pallor, making OCT useful for detecting subclinical optic neuropathy in SPG7 patients (47).

#### Transcranial Magnetic Stimulation

HSP patients with an increased score on the Ashworth spasticity scale have a shorter cortical silent period relative to normal controls (48). This is a measure of supraspinal or intracortical inhibitory pathways, and not degeneration in the corticospinal tract axons (48). Other measures that were significantly different between HSP and controls were tibialis anterior motor threshold, total motor conduction time (TMCT), central motor conduction time (CMCT), and motor-evoked potential (MEP) amplitude; however, these did not correlate with the Ashworth spasticity scale (48). One study found 37% of HSP patients had prolonged CMCT to lower extremities, and CMCT to the legs correlated with total SPRS score (*r* = 0.176; *p* < 0.028) and spastic subscore (*r* = 0.241, *p* < 0.005); pathologic CMCT to legs correlated with disease duration (*r* = 0.231; *p* < 0.009) (27). SPG4 CMCTs were normal in both arms (5.1 ± 1.5 ms) and legs (14.1 ± 3.9 ms), while non-SPG4 HSP patients' CMCTs were significantly longer in arms (6.9 ± 2.6 ms) and legs (18.2 ± 7.3 ms). Across subgroups of SPG4 patients, CMCT to legs in those with missense mutations were shorter (10.5 ± 1.3 ms) than in those with SPG4 due to premature stop codons, exon deletions, or splice site mutations (13.9 ± 3.7 ms) (27).

#### Completed Clinical Trials for HSP

A number of clinical trials of pharmacological agents have been tested in HSPs, as outlined in Table 1 and described below.

**Table 1:** Completed clinical trials for HSPs.

**Intervention**	**Outcome measures**	**Results**	**PubMed ID**	**Patients (*n*)**	**Controls (*n*)**	**Study design**
Atorvastatin at 40 mg/day for 9 weeks in adults and children under 18 received 20 mg/day (13)	Primary outcome: Change of 27-OHC in serum after 9 weeks. Secondary outcomes: change of 27-OHC in CSF, reduction of 24-OHC, 25-OHC and 3β-CA levels in serum and CSF	Statistically significant reduction in serum 24S-OHC, 25-OHC, and 27-OHC. No statistically significant change in other measures tested, notably in CSF.	29126212	7 enetically confirmed SPG5 patients	7 genetically confirmed SPG5 patients	Randomized, placebo-controlled trial
Atorvastatin 20 mg b.i.d, CDCA 500 mg b.i.d, resveratrol 40 mg b.i.d; three periods of 2 months of treatment separated by a 4-month washout. (14)	Primary outcome measure: Plasma 27-OHC levels Secondary outcome measures: plasma 25-OHC levels, plasma oxysterols to total cholesterol ratio, serum bile acids profile, safety profile of study drugs	Statistically significant reduction in plasma 27-OHC and 24S-OHC and cholesterol with Atorvastatin. Plasma oxysterols were not altered by CDCA or resveratrol.	29228183	12 genetically confirmed SPG5 patients	Three-period crossover study	Three-period, three-treatment crossover study.
Intrathecal baclofen (32)	Spasticity measured on the Ashworth scale (30), walking ability assessed with Gillette Functional Assessment Questionnaire (31)	Reduction of lower limbs' spasticity as measured by modified Ashworth scale from 2.6 (±0.8) to 0.7 (±0.9) (*p* = 0.000). Walking ability improved in modified version of functional walking scale of Gillette Functional Assessment questionnaire from 5.9 (±1.7) to 7.4 (±2.0) (*p* = 0.001).	24973568	14 patients clinically diagnosed with HSP	0	Open-label, prospective study with 14/16 patients who responded to baclofen trial.
Intrathecal baclofen pump for 3 months of continuous infusion (49)	Ashworth scale, reflexes, and spasm score 3 months after continuous infusion of baclofen pump	Ashworth scale decreased from 3.96 ± 0.81 to 1.92 ± 0.58, reflexes decreased from 4.25 ± 0.45 to 2.00 ± 0.0, and spasm score decreased from 2.50 ± 0.55 to 1.83 ± 0.41.	1514885	3	0	Open label
Botulinum toxin type A (500 MU in each leg with calf muscle tone MAS1 or 750 MU in each leg with calf muscle tone MAS2 with injections over the 3 heads of triceps surae and heads of gastrocnemius) and daily stretching exercises for 18 weeks (26)	Primary outcome: change in 10 m comfortable gait velocity gait velocity(28, 29) 8 weeks after injections (averaging three trials) and calf muscle tone Secondary outcome: change in SPRS (24, 25); change in Ashworth spasticity scale (30); Medical Research Council (MRC) strength scale; Quantitative Muscle Assessment (QMA) fixed myometry testing; brief pain inventory scale, modified fatigue impact scale; Fugl-Meyer scale (33) and timed up and go testing (34).	Mean comfortable gait velocity increased from 0.90 m/s (*SD* = 0.18) to 0.98 m/s (*SD* = 0.20) at 4 weeks after treatment which persisted to 18 weeks after treatment. Calf muscle tone decreased from median 2, range 1–2 to median 1, range 0–1 at 4 weeks, which partially persisted at 18 weeks with median 1, range 0–2.The change in MRC, QMA, maximum gait velocity, Activities-specific Balance Confidence Scale change post treatment was not statistically significant. Other functional measure remained unchanged after treatment.	25325386	15 autosomal dominant pure HSP genetically proven SPG4, 3A and 8, or based on family history	0	Open label
Dalfampridine 10 mg twice daily for 2 weeks (50)	The Timed 25-foot walk test (51), the Spastic Paraplegia Rating Scale (SPRS) (24) and the 12-item Multiple Sclerosis Walking Scale (MSWS-12) (52) performed before and after treatment.	SPRS score improved from 21 ± 9.6 to 19.1 ± 9.6 (*p* = 0.0195) and MSWS-12 improved from 70.3 ± 19.6 to 61 ± 22.7 (*p* = 0.0429)	25808501	12 patients: SPG4 (*n* = 6); SPG7 (*n* = 1); SPG10 (*n* = 2); adrenomyeloneuropathy (*n* = 2), history of childhood onset pure spastic paraplegia (*n* = 1)	0	Prospective, uncontrolled, proof-of- concept open trial.
Gabapentin (53) Placebo or gabapentin 2400 mg daily titrated up to 4000 mg daily over 10 days, maintained for 2 months, then washout of 10 days and drug free for 1 month, followed by crossover to treatment or placebo.	MS impairment scale (35), Visual Analog Scale score of current life quality, paired transcranial magnetic stimulation	No statistically significant differences comparing gabapentin with placebo. No evidence of altered intracortical excitability.	17539946	10 SPG4 patients (6/10 genetically confirmed)	Crossover study	Prospective double-blind, randomized, placebo-controlled, crossover trial
L-Threonine (54) 7 patients took 1.5 g t.i.d and 9 patients took 2 g t.i.d and 2 additional patients took both doses by completing the double-blind study twice.	Spasticity variables: Leg strength; leg tone, leg deep tendon reflexes, clonus, walk, hop on foot, run	Mean spasticity score went from 1.64 ± 0.17 to 1.40 ± 0.16 on L-threonine and from 1.56 ± 0.16 to 1.54 ± 0.18 on placebo. However, clinical assessments and patients' self-evaluations of response did not show significant differences.	1742749	18 familial spastic paraparesis patients	Double-blind, crossover design	Double-blind, crossover design; 2 treatment periods lasting 2 weeks interrupted by 2-weeks washout period
22 patients treated with 60 mg methylphenidate daily for 6 months (55)	Gait analysis by computerized three-dimensional infrared movement analysis system at baseline, 6 weeks of treatment and 6 months of treatment. Ashworth spasticity scale (30); MRC scale	No statistical difference between baseline and 6 weeks and 6 months of methylphenidate treatment as assessed by Ashworth spasticity scale and MRC Scale. A small increase in gait velocity from 2.56 ± 1.07 km/h to 2.85 ± 0.97 km/h, *p* = 0.019), which decreased back to baseline at 6 months.	16705687	22 SSP or HSP patients based on clinical dianosis. 6/22 were genetically confirmed to have SPG4 mutations.	0	Open-label study with a longitudinal follow-up

##### Atorvastatin 40 mg/day (20 mg/day in children) for SPG5 (13)

This study targeted a specific sub-type of HSP, SPG5, which is a rare autosomal recessive HSP due to mutation in oxysterol-7α-hydroxylase protein CYP7B1, which degrades side chain oxidized cholesterols. It was hypothesized that reducing cholesterol levels with atorvastatin would reduce toxic side-chain oxidized cholesterols, particularly 27-OHC, and while serum levels were lowered, CSF levels were not. There was no clinical benefit noted in the short time period of treatment (9 weeks).

##### Atorvastatin 20 mg BID, chenodeoxycholic acid (CDCA) 500 mg BID, resveratrol 40 mg BID in SPG5 (14)

In this study of SPG5, the primary outcome measure was plasma 27-OHC levels and secondary outcome measures included plasma 25-OHC levels, plasma oxysterols to total cholesterol ratio, a serum bile acids profile, and the safety profile of study drugs. The trial was designed such that a total of 12 SPG5 patients were treated for three periods of 2 months with each agent, separated by a 4-month washout. There was a statistically-significant reduction in plasma 27-OHC, 24S-OHC and cholesterol levels with atorvastatin. However, plasma oxysterols were not altered by CDCA or resveratrol.

##### Baclofen pump trial (49)

Patients were given either normal saline or 50 μg of baclofen intrathecally, and those who had an average drop of two points in muscle tone and reflexes score were offered implantation of a baclofen pump and then monitored after 3 months of therapy. Measures of response of three patients after 3 months of therapy were: the Ashworth scale (0–5), the spasm score (0–4 with 0 no spasms, 1 mild spasms by stimulation, 2 infrequent spasms less than once per hour, 3 spasms more than once per hour, and 4 spasms occurring more than ten times an hour), and reflex score (0–5). They found that with a baclofen pump for 3 months, the Ashworth score decreased from 3.96 ± 0.81 to 1.92 ± 0.58, reflexes decreased from 4.25 ± 0.45 to 2.00 ± 0.0, and the spasm score decreased from 2.50 ± 0.55 to 1.83 ± 0.41.

##### Intrathecal baclofen pump trial (32)

In this study, 14 HSP patients had baclofen pumps placed finding a reduction of lower limb spasticity as measured using a modified Ashworth scale from 2.6 ± 0.8 to 0.7 ± 0.9 (*p* = 0.000) and that patients' walking ability improved as assessed in a modified version of functional walking scale of the Gillette Functional Assessment questionnaire from 5.9 ± 1.7 to 7.4 ± 2.0 (*p* = 0.001).

##### Botulinum toxin type A and stretching in HSP (26)

A clinical trial tested botulinum toxin type-A [500 mouse LD_50_ units (MU) in each leg with calf muscle tone MAS1 or 750 MU in each leg with calf muscle tone MAS2, with injections over the 3 heads of triceps surae and heads of gastrocnemius] and daily stretching exercises for 18 weeks in HSP patients (26). The primary outcome measure was a change in baseline of 10-m comfortable gait velocity (28, 29) measured at 4 weeks (peak effect time) and 18 weeks (residual effects of botulinum toxin type A) after injections, with an average of three trials used as final measure at each time point. Secondary outcome measures included change from baseline in SPRS (24, 25), change in baseline in Ashworth spasticity scale (30) of adductors and triceps surae, change from baseline in muscle strength, change from baseline in visual analogic scale of pain, brief pain inventory scale, and modified fatigue impact scale. The authors also addressed the Fugl-Meyer scale (33) and timed up-and-go testing (34). Interestingly, it was comfortable gait velocity that increased post-botulinum toxin type A and stretching, not maximum gait velocity. Besides tone, other measures did not statistically differ post-treatment.

##### Dalfampridine 10 mg twice daily for 2 weeks (50)

In this prospective, uncontrolled, open label trial, 12 patients with different forms of HSP were treated with dalfampridine 10 mg twice daily for 2 weeks. While there was no control group, the total SPRS score improved from 21 ± 9.6 to 19.1 ± 9.6 (*p* = 0.0195), and the MSWS-12 improved from 70.3 ± 19.6 to 61 ± 22.7 (*p* = 0.0429). Given the lack of a control group, the authors noted that a placebo effect could not be ruled out.

##### Gabapentin 2,400 mg daily titrated to 4,000 mg daily (53)

In this prospective double-blind, randomized placebo-controlled crossover trial, patients were given placebo or gabapentin 2,400 mg daily titrated up to 4,000 mg daily over 10 days, maintained for 2 months; next, a washout of 10 days and drug-free period for 1 month were followed by crossover to treatment or placebo. Of note, 2/10 patients had gabapentin levels below 20 mmol on testing, indicating possible non-compliance with therapy. Patients were assessed using the MS Impairment Scale (35), Visual Analog Scale score of current life quality, and paired transcranial magnetic stimulation. Overall, no significant differences were noted between gabapentin treatment and placebo arms.

##### L-Threonine at 4.5 or 6.0 g/day (54)

One early clinical trial tested L-threonine in 18 patients with familial spastic paraparesis in a double-blind crossover protocol for 2 weeks, at doses of 4.5 or 6.0 g/day. The rationale was to increase the inhibitory glycinergic tone of spinal cord interneurons with L-threonine, since glycine has limited blood-brain barrier penetration. While the treatment increased plasma and CSF levels of L-threonine with statistical significance, glycine levels in blood and CSF were not. While clonus and deep tendon reflexes improved on L-threonine, overall clinical assessments and patients' self-evaluations showed no differences between threonine and placebo treatment.

##### Methylphenidate 60 mg daily for 6 months (55)

In this open-label study with a longitudinal follow-up, 22 patients were treated with 60 mg methylphenidate daily for 6 months. While 6 of 22 patients were genetically confirmed to harbor SPG4 mutations, the remaining 16 patients had a clinical diagnosis of HSP or a sporadic form (SSP). A small increase in gait velocity from 2.56 ± 1.07 to 2.85 ± 0.97 km/h (*p* = 0.019) reverted to baseline at 6 months; this could be attributed to possible placebo effect. One significant limitation in this study was the absence of a placebo group. Additional assessment measures showed no statistical difference between baseline and 6 weeks and 6 months of methylphenidate treatment as assessed by Ashworth spasticity scale and Medical Research Council Scale, cadence (steps/min), and stride length.

### Case Reports of Therapeutic Agents

In a family with SPG8, an affected mother and daughter were started on L-DOPA (Co-Careldopa 25/100 mg); the mother reported responses in spasms and sensory symptoms in feet (which included burning), and the daughter experienced improvement in spasticity (56). A case study of SPG11 also reported some response of parkinsonism to levodopa (57).

### Clinical Trials of Non-pharmacological Treatments

One group bypassed the corticospinal tract in treatment using a startle response. They tested a startling acoustic stimulus (SAS) in 12 HSP patients (8 SPG4, 1 SPG8, and 3 unknown) as well as 12 healthy controls; voluntary ankle dorsiflexion reaction time onset latency was 146 ± 23 ms in HSP patients compared with 127 ± 15 ms in controls without a SAS, but administration of a SAS resulted in HSP patients having onset latency of 89 ± 20 ms compared to controls 91 ± 12 ms. The authors hypothesized that SAS released a subcortically-stored motor program relayed through the reticulospinal tract that improved response similar to normal controls (58). Additionally, 10 weeks of hydrotherapy has been tried in 9 HSP patients, with improvement in baseline velocity from 0.85 ± 0.22 to 0.94 ± 0.27 m/s (59). A clinical trial testing 6 weeks of robotic gait training in 13 uncomplicated HSP patients showed improvement in balance, walking speed, and endurance both after completing the treatment and at 2 months follow up (60).

## Discussion

### Completed Trials

A clear conclusion that can be garnered from clinical trials completed in HSPs is that, apart from cholesterol modifying therapy in SPG5 reported in 2017 and 2018 (13, 14), molecularly-targeted therapies have been noticeably absent. Other clinical trials have targeted clinical manifestations of HSPs, specifically spasticity. This may be due to an earlier focus on the symptomatic treatment of spasticity, as molecular and genetic understanding of HSP was limited. More recently, clinical trials targeting the molecular pathways of HSP have become more practical. Interestingly, clinical trial designs directed toward treating the symptom of spasticity have not extended to evaluating other common symptoms such as urinary incontinence.

Clinical trial design also has evolved more generally, and this is particularly important in rare diseases such as the HSPs with limited patient numbers. Many of the clinical trials presented here are open-label, unblinded studies; this raises the specter of placebo effect and overall interpretability of the findings. Better trial designs have been increasingly utilized, including double-blinded and cross-over studies. Utilizing the same patients in different arms of a study by cross-over, separated by drug washout, can facilitate clinical trial design for rare diseases.

Careful attention must be paid to whether physiological effects of a drug might persist beyond the washout period. Duration of therapy is also a notable challenge in clinical trials of HSP. While 2 months of CDCA treatment did not yield significant results (14), a case study of 5 years of CDCA treatment showed a marked reduction in 27-OHC levels in a SPG5 patient (61). Assessment of patient compliance by testing serum or urine for drug levels also is important in clinical design. In the clinical trial of gabapentin, serum levels were found to be low in 2/10 patients, raising questions of compliance (53). Given a starting dose of 2,400 mg daily titrated up to 4,000 mg daily over 10 days, it is conceivable that the side effect profile of gabapentin could have impacted patient compliance, particularly when initiated at a relatively high dose. Notably, there are several instances of differences between serum and CSF levels of diagnostic markers, specifically in the L-threonine (54) and atorvastatin trials (13). Care should be taken in attempts to extrapolate plasma biomarkers for HSP, as CSF levels could differ. Indeed, it is possible to therapeutically impact a biomarker without influencing the underlying disease process, and clinical assessment measures are critical in trials.

### Future Trials

The future for innovative clinical trial design in the HSPs is encouraging, since we are learning progressively more about the underlying molecular mechanisms of disease. This knowledge has helped to provide molecular targets, biomarkers, animal assessment measures and human clinical measures to assess the therapeutic response for future clinical trials. While this review identifies multiple assessment measures relied upon to quantify response, a key focus on selecting measures that demonstrate effects on how one feels, functions, or survives would likely be more favorably received at the FDA.

### HSPs as Rare Diseases

The Orphan Drug Act of 1983 defines rare diseases as those affecting <200,000 people in the United States (62). Since HSPs occur in ~1.8 per 100,000 people (1), and given a population of about 325.7 million in the US, this translates to nearly 6,000 patients, rendering HSPs a rare disease, but one with sufficient numbers to perform meaningful trials. The individual genetic forms, of course, are rarer still. Information from natural history studies that evaluate exploratory biomarkers can help to design an efficient drug development pathway. Natural history biomarkers can also provide an understanding of subpopulations as well as help select outcome measures that are more specific or sensitive to changes.

The FDA encourages natural history studies to include a spectrum of disease severity and phenotypes over a significant duration. Prior to first-in-human studies, the FDA requires toxicology from *in vitro* experiments, animal studies, or both; for toxicology profiles, healthy animals are preferred over animal models of disease. Study endpoints should be optimized for validity, reliability, feasibility, resistance to bias, ability to detect change, relationship to meaningful symptoms or function, and clinical interpretability (63).

### Biomarkers

Biomarkers may be diagnostic (categorizes disease), prognostic (risk of disease / progression), predictive (response to treatment), or pharmacodynamic (biological response to intervention) (64). The FDA has provided guidance regarding biomarker use for drug development (64, 65), and biomarkers can be used to help in subject selection for clinical trials (65) in certain situations (e.g., enrichment or prognostic biomarkers). Given the large number of genetic causes of HSP, genotyping subjects prior to clinical trials will likely be important. A biomarker can also help assess disease state (65). Preliminary work has shown that CMCT is a prognostic biomarker (27); however, the question of whether CMCT could serve as a pharmacodynamic biomarker [as CMCT does in vitamin B_12_ deficiency (66)] remains unanswered. A search for additional biomarkers that may have higher specificity and sensitivity is underway. Preliminary work is studying miRNAs (38, 39), measures of oxidative stress (3), and levels of acetylated α-tubulin (6), among others. While *p*-values are important, sensitivity and specificity are also critical in biomarker development. Biomarkers can help assess mechanism of action and the mechanism of therapeutic effect (65). Biomarkers can also inform dose optimization, particularly in animal models, to determine no observed effect level (NOEL) and no observed adverse effect level (NOAEL) (65). Animal study measures discussed in this review could be helpful in generating these values, with potential extrapolation to human trials. A biomarker can also help monitor a drug's response, particularly safety and efficacy, in attempts to maximize drug efficacy and minimize adverse reactions (65).

## Conclusions

In this review, we have set out to provide a roadmap for future clinical trials for the HSPs. Promising therapeutic targets include oxidative stress, microtubule dynamics, genetic rescue strategies, and for more specific subsets of HSPs, autophagy/endolysosomal function and cholesterol metabolism. We have reviewed animal models available to test potential agents on as well as animal assessment measures available to evaluate effect. Blinded experiments on animals will be critical for determining which therapeutics should be advanced to human trials. We have described a number of human clinical assessment tools available to evaluate the physical effects of HSPs and emphasize that the FDA looks for a positive effect on how one feels, functions, or survives as a clinical endpoint. With increasing knowledge of the genetic and cellular basis of HSPs, there will be more opportunities for clinical trial development. Biomarkers, animal models and assessment measures, electrophysiology, and clinical assessment measures continue to be refined, expanding our knowledge of the natural history of HSP. We are hopeful that application of these insights and clinical trial designs will usher in novel clinical trials in this rare disease population.

## Author Contributions

BT performed research and wrote the manuscript. DH and CB edited the manuscript for important intellectual content.

### Conflict of Interest Statement

The authors declare that the research was conducted in the absence of any commercial or financial relationships that could be construed as a potential conflict of interest.
